# Taxonomic structure and functional association of foxtail millet root microbiome

**DOI:** 10.1093/gigascience/gix089

**Published:** 2017-09-05

**Authors:** Tao Jin, Yayu Wang, Yueying Huang, Jin Xu, Pengfan Zhang, Nian Wang, Xin Liu, Haiyan Chu, Guang Liu, Honggang Jiang, Yuzhen Li, Jing Xu, Karsten Kristiansen, Liang Xiao, Yunzeng Zhang, Gengyun Zhang, Guohua Du, Houbao Zhang, Hongfeng Zou, Haifeng Zhang, Zhuye Jie, Suisha Liang, Huijue Jia, Jingwang Wan, Dechun Lin, Jinying Li, Guangyi Fan, Huanming Yang, Jian Wang, Yang Bai, Xun Xu

**Affiliations:** 1 BGI-Qingdao, Qingdao, 266510, China; 2 China National Genebank-Shenzhen, BGI-Shenzhen, Shenzhen, 518083, China; 3 Citrus Research and Education Center, Department of Microbiology and Cell Science, IFAS, University of Florida, Lake Alfred, FL, USA; 4 BGI Education Center, University of Chinese Academy of Sciences, Shenzhen, 518083, China; 5 State Key Laboratory of Soil and Sustainable Agriculture, Institute of Soil Science, Chinese Academy of Sciences, East Beijing Road 71, Nanjing, 210008, China; 6 Laboratory of Genomics and Molecular Biomedicine, Department of Biology, University of Copenhagen, DK-2100, Copenhagen, Denmark; 7 BGI Millet Co., Ltd, Shenzhen, 518083, China; 8 James D. Watson Institute of Genome Sciences, Hangzhou 310058, China; 9 State Key Laboratory of Plant Genomics, Institute of Genetics and Developmental Biology, Chinese Academy of Science, Beijing, 100101, China; 10 Centre of Excellence for Plant and Microbial Sciences (CEPAMS), Institute of Genetics and Developmental Biology, Chinese Academy of Science & John Innes Centre, Beijing, 100101, China; 11 The University of Chinese Academy of Sciences, Beijing, 100049, China

**Keywords:** root, microbiome, 16S rRNA, *Setaria italica*, productivity

## Abstract

The root microbes play pivotal roles in plant productivity, nutrient uptakes, and disease resistance. The root microbial community structure has been extensively investigated by 16S/18S/ITS amplicons and metagenomic sequencing in crops and model plants. However, the functional associations between root microbes and host plant growth are poorly understood. This work investigates the root bacterial community of foxtail millet (*Setaria italica*) and its potential effects on host plant productivity. We determined the bacterial composition of 2882 samples from foxtail millet rhizoplane, rhizosphere and corresponding bulk soils from 2 well-separated geographic locations by 16S rRNA gene amplicon sequencing. We identified 16 109 operational taxonomic units (OTUs), and defined 187 OTUs as shared rhizoplane core OTUs. The β-diversity analysis revealed that microhabitat was the major factor shaping foxtail millet root bacterial community, followed by geographic locations. Large-scale association analysis identified the potential beneficial bacteria correlated with plant high productivity. Besides, the functional prediction revealed specific pathways enriched in foxtail millet rhizoplane bacterial community. We systematically described the root bacterial community structure of foxtail millet and found its core rhizoplane bacterial members. Our results demonstrated that host plants enrich specific bacteria and functions in the rhizoplane. The potentially beneficial bacteria may serve as a valuable knowledge foundation for bio-fertilizer development in agriculture.

## Introduction

The root surface sets the environment for complex interactions among soil, the host plant, and microbes [1]. The root microbiota (rhizosphere, rhizoplane, and endophytic bacteria) mainly derive from surrounding soil and are influenced by geographical locations, nutrient status, and host genotype [1–9]. These microbes play pivotal roles in plant productivity, nutrient uptakes, and disease resistance. Observations from multiple research teams imply that plants selectively “cultivate” specific and potentially beneficial microbes through root exudates and deposits, which act as a carbon source and nutrients for microbial growth, as well as altering soil pH structure [10, 11]. However, how the root microbiota influence plant growth and yield remains largely unknown. Comprehensive association studies between the root microbiota and crop traits are needed to identify beneficial or harmful microbes.

Foxtail millet (*Setaria italica*) is an important crop in arid and semiarid regions due to its water use efficiency and drought tolerance [12]. It is crucial to understand the genetic and environmental factors of foxtail millet; 2540 foxtail millet cultivars with the phenotypic traits and genomes were deposited in the China National Gene Bank-Shenzhen [13–15].These collections facilitate a deep understanding of how the genotypes and root microbiota affect the foxtail millet growth, development, and yield.

In this study, we sequenced the rhizosphere and rhizoplane bacterial microbiota in 1219 foxtail millet cultivars, which were grown in 2 far-separated fields in China, Yangling and Zhangjiakou. We evaluated the effects of geographic location and microhabitat on root bacterial communities and predicted root bacterial functions according to their taxonomy. We performed systematic association analysis between rhizoplane bacteria and foxtail millet productivity traits and identified specific bacterial taxa correlated with host plant productivity. Our work provides a basis for potential agricultural improvement of foxtail millet by root microbiota modification.

### Data description

**Figure 1:**
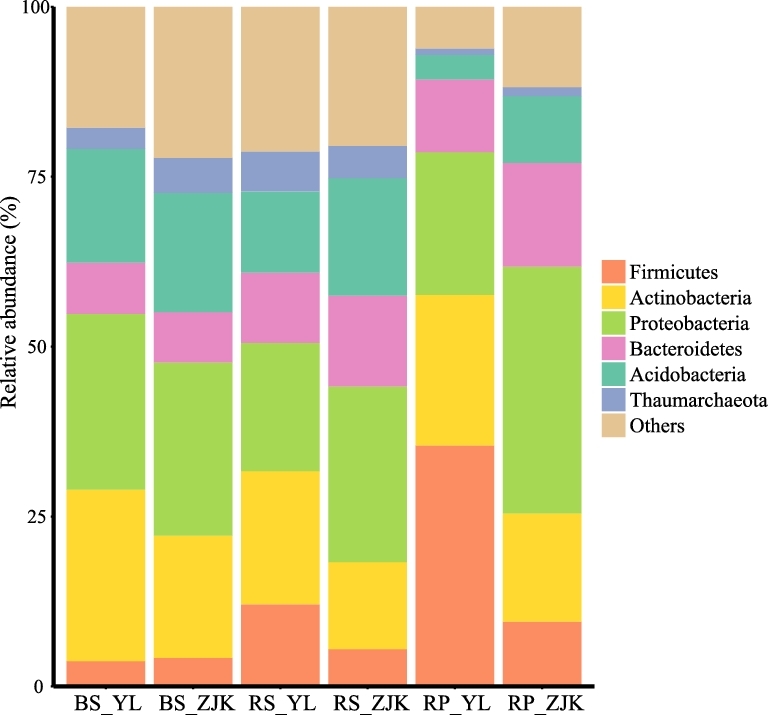
Dominant bacterial phyla detected in foxtail millet root compartments and bulk soils. BS: bulk soil; RP: rhizoplane; RS: rhizosphere; YL: Yangling; ZJK: Zhangjiakou.

The root microbiota of foxtail millet remains largely unknown. In this study, we collected rhizosphere, rhizoplane, and corresponding unplanted bulk soil samples from the foxtail millet cultivars in 2 well-separated locations in China. We recorded 12 foxtail millet traits related to growth and productivity (Tables S1 and S2). We sequenced the V4-V5 region of the 16S rRNA gene for 2882 samples, which yielded 98 750 591 high-quality reads for subsequent analysis, an average of 34 264 sequences per sample. After discarding low-abundance and non-bacterial operational taxonomic units (OTUs), we obtained 16 109 OTUs, 2998 OTUs per sample (Table S3), with 97% similarity of the entire V4-V5 region; 81.3% of the OTUs could be assigned to 34 bacterial phyla, mainly including Acidobacteria, Actinobacteria, Bacteroidetes, Firmicutes, and Proteobacteria. In total, 624 genera belonging to 254 families were recorded from these samples. Rhizoplane and rhizosphere contained a large number of total OTUs, and only 30 and 26 OTUs per sample showed relative abundances higher than 0.5% on average. Bulk soil samples contained the lowest number of total OTUs because of the small sample size. A summary of the data is in Table 1. Taxa at the phylum level of rhizosphere and rhizoplane microbiota are shown in Fig. 1 and Table S4. The sequencing data have been deposited in the European Nucleotide Archive (accession PRJEB16061).

**Table 1:** Summary of samples and OTUs assigned to different taxonomic levels

					% of OTUs assigned to		
Sample group	Sample number	Average reads/sample	Average OTUs/sample	No. of OTUs	Genus	Family	Phylum
BS.YL	8	23 473	2980	8132	59.7	68.8	92.2
RS.YL	1219	33 373	3333	15 676	62.2	78.8	92.8
RP.YL	1219	36 396	2479	15 110	54.1	88.4	97.7
BS.ZJK	8	54 756	4414	9426	56.7	64.3	88.6
RS.ZJK	214	26 218	3555	14 696	63.7	73.8	92.8
RP.ZJK	214	34 879	3433	14 558	65.6	80.1	95.3
Total	2882	34 264	2998	16 109			

YL: Yangling; ZJK: Zhangjiakou; BS: bulk soil; RS: rhizosphere; RP: rhizoplane.

## Results

### Main factors shaping the root-associated bacterial composition

The rarefaction curves by the Chao1 index indicated that the sequencing depth was sufficient to cover the bacterial diversity within individual samples (Fig. S1a). Additionally, the large sample size allowed us to capture the bacterial diversity in rhizosphere and rhizoplane compartments in each site because the observed OTU numbers reached saturation (Fig. S1b). Yangling and Zhangjiakou samples shared 14 992 OTUs, which make up the majority of OTUs from Yangling (15 825 OTUs) and Zhangjiakou (15 276 OTUs). Consistent with previous observations [3, 4], the α-diversity decreased from rhizosphere to rhizoplane in both fields (Wilcoxon signed rank test, *P* < 0.001) (Fig. S1c and d).

**Figure 2:**
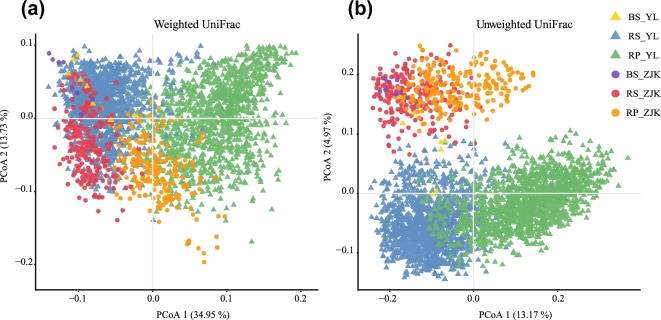
Principal coordinate analysis of foxtail millet root microbiota based on weighted (**a**) and unweighted (**b**) Unifrac matrices indicating that microhabitat is the largest separation factor (PCoA1) and geographic location is the second (PCoA2). BS: bulk soil; RP: rhizoplane; RS: rhizosphere; YL: Yangling; ZJK: Zhangjiakou.

We evaluated the influence of geographical locations and microhabitat on foxtail millet root microbiota structure using UniFrac distance. Rhizoplane and rhizosphere microbiota separated along the first principal coordinate (Fig. 2). The second principal coordinate was explained by geographical locations. These results demonstrated that microhabitat and the geographic locations contributed the majority of microbiota variations in foxtail millet roots. A partial canonical analysis of principal coordinates (CAP) revealed that microhabitat explained the largest proportion (26.05%, *P* < 0.001) of the variation in β-diversity, followed by geographical locations (5.81%, *P* < 0.001) (Fig. S2). This pattern was recapitulated by PERMANOVA based on UniFrac distances (Table S5).

The similarity between paired rhizosphere and rhizoplane microbiota in Zhangjiakou was significantly higher than that in Yangling (Sørensen–Dice index and Morisita-Horn's index, Wilcoxon signed rank test, *P* < 0.005) (Table 2). According to the 3-step enrichment model [10], our results demonstrated that the influence of the host to the rhizosphere microbiota was different in these 2 sampling sites, which may be due to the corresponding soil characteristics.

**Table 2:** Community structure similarity of paired rhizoplane and rhizosphere microbiota for each cultivar was assessed by Sørensen–Dice index and Morisita-Horn's index

			Differences in variation of community structure tested using Wilcoxon signed rank test
Index	The mean value of paired samples in YL	The mean value of paired samples in ZJK	*P* value
Sørensen–Dice	0.537	0.552	0.0042
Morisita-Horn's	0.393	0.517	1.44e-11

YL: Yangling; ZJK: Zhangjiakou.

### Core rhizoplane bacteria

**Figure 3:**
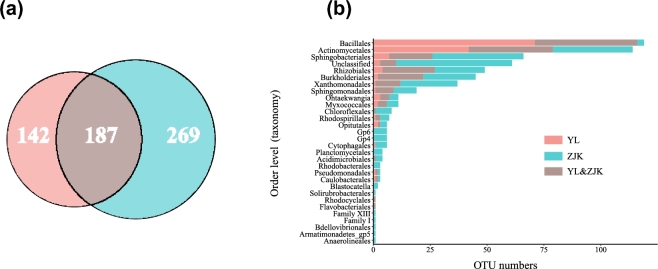
Characterization of the core rhizoplane microbiota. (**a**) Venn diagram depicting the number of shared and specific OTUs of the core rhizoplane OTUs from both fields. (**b**) Taxonomic distribution of the core rhizoplane OTUs at the order level. The shared core rhizoplane OTUs from YL and ZJK were colored with brown, and the specific core OTUs from YL and ZJK were colored with red and blue, respectively. BS: bulk soil; RP: rhizoplane; RS: rhizosphere; YL: Yangling; ZJK: Zhangjiakou.

We defined 329 and 456 core rhizoplane OTUs from Yangling and Zhangjiakou rhizoplane microbiota, respectively, which were present in more than 80% of the samples in each group and were statistically enriched in rhizoplane compared with rhizosphere (Wilcoxon signed rank test, Benjamini-Hochberg adjusted *P* < 0.01). These core rhizoplane microbes represented 63.97% and 51.17% of Yangling and Zhangjiakou rhizoplane microbiota, respectively. Among the shared 187 core rhizoplane OTUs from both locations (Fig. 3a), 144 OTUs belonged to Bacillales, Actinomycetales, Rhizobiales, Burkholderiales, and Sphingobacteriales (Fig. 3b). More importantly, 15 OTUs in above 5 orders were present in all rhizoplane samples and occupied on average 20.02% and 25.63% of sequencing reads from Yangling and Zhangjiakou rhizoplane samples, respectively (Table S6).

The core rhizoplane microbes in Zhangjiakou were more evenly distributed in taxonomic groups than those in Yangling (Fig. 3b). The most dominant order in Yangling core rhizoplane microbes was Bacillales, representing 116 of 329 core OTUs. The average relative abundance of Bacillales in Yangling rhizoplane samples was 32.72%, 2-fold higher than that of the second dominant order, Actinomycetales. By contrast, the main core rhizoplane microbes of Zhangjiakou were composed of Actinomycetales (8.92%), Burkholderiales (8.13%), Bacillales (6.95%), and Sphingobacteriales (6.80%).

Compared to bulk soil and rhizosphere samples, the main depleted bacterial phylum in rhizoplane samples was Acidobacteria. The relative abundance of Acidobacteria decreased from bulk soil (16.74%) to rhizosphere (11.95%) and rhizoplane microbiota (3.63%) in Yangling (Wilcoxon rank test, *P* < 0.005). A similar pattern was found in Zhangjiakou samples (Fig. 1). There was no OTU assigned to Acidobacteria in the shared core rhizoplane microbes. The previous studies also revealed that Acidobacteria was widespread in different types of soils but was not present in the plant root [3, 8, 16].

### Root bacteria correlated to foxtail millet growth and productivity

We first evaluated the correlation between rhizoplane microbiota and foxtail millet traits using the Yangling dataset because of its large sample size. We found rhizoplane microbiota remarkably correlated with the panicle weight of the main stem, grain weight per plant, top second leaf width, main stem width, and panicle diameter of the main stem (Benjamini-Hochberg adjusted *P* < 0.05, PERMANOVA and the Mantel test) (Table S7). Meanwhile these traits were correlated to each other, and together they represent the general plant performance (Fig. S3a).

**Figure 4:**
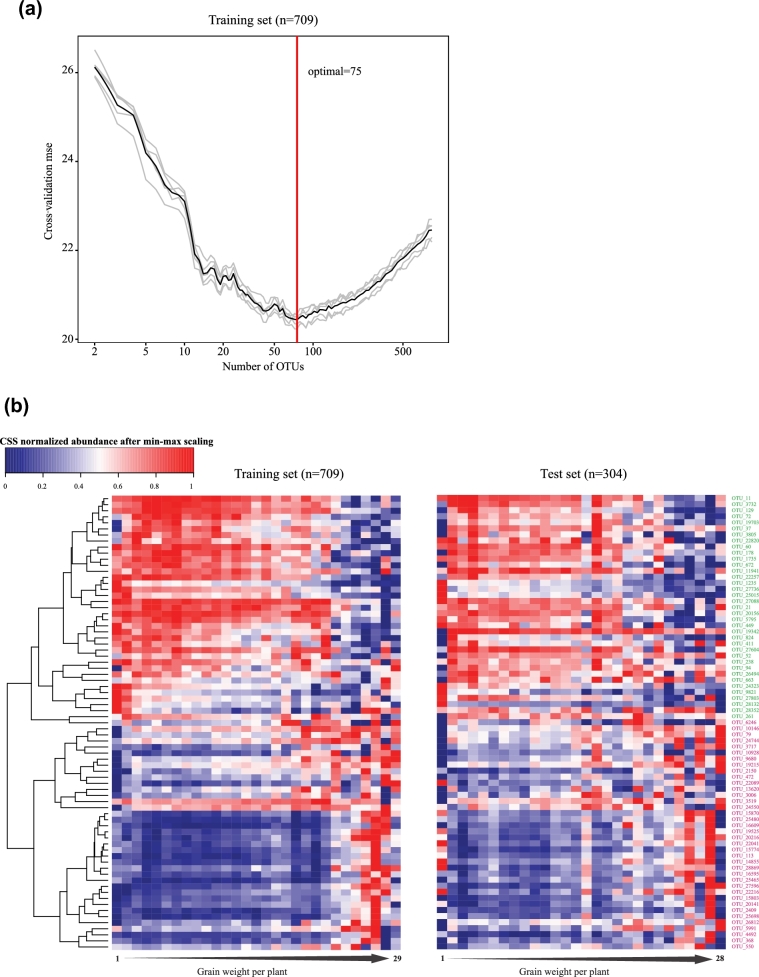
Bacterial marker OTUs correlated with foxtail millet general health in Yangling. (**a**) A random forest model was applied to regress the rhizoplane bacterial OTUs against grain weight. Five repeats of 10-fold cross-validation in the training set (n = 709) resulted in 75 marker OTUs to predict grain weight of foxtail millet. (**b**) Heat map and hierarchical clustering of the mean of marker OTUs’ relative abundance against grain weight. The samples were grouped according to grain weight (range from 1–30g); e.g., grain weight per plant of less than 2 g was classified into the first group, less than 3 g for the second group, and so on. The left heat map was plotted with training set samples and the right from the test set samples. OTUs negatively and positively correlated with the yield were marked with different colors; negative: red; positive: green.

We focused on the correlation between the rhizoplane microbiota and grain weight per plant, which is a typical trait to reflect the theoretical yield of foxtail millet [17]. We trained a random forest model with 839 OTUs (occurrence frequency ≥ 0.3 and adjusted *P* < 0.05, Spearman's rank correlation test) in 709 rhizoplane samples. Five repeats of 10-fold cross-validation (that is, 50 tests) in the training set resulted in the selection of 75 OTU markers for grain weight per plant (Fig. 4a). A correlation (*R*^2^ = 0.31) between OTUs and grain weight per plant was determined in the out-of-the-bag samples of the training set. A similar correlation (*R*^2^ = 0.293) was also found in the test set (*n* = 304) (Fig. 4b); 38 and 37 marker OTUs were positively and negatively correlated with the productivity, respectively. The relative abundance distribution of all the marker OTUs varied in different productivity groups of foxtail millets (Fig. 4b). In addition, correlations between these marker OTUs and other phenotypes such as the panicle weight of the main stem, panicle diameter of the main stem, grain number per spike, and top second leaf width were also observed (Fig. S3b). These results indicated that foxtail millet productivity results from the combination of plant genetics and rhizoplane microbiota. Plants may adapt to the environments by actively or passively regulating beneficial and harmful root microbes.

**Figure 5:**
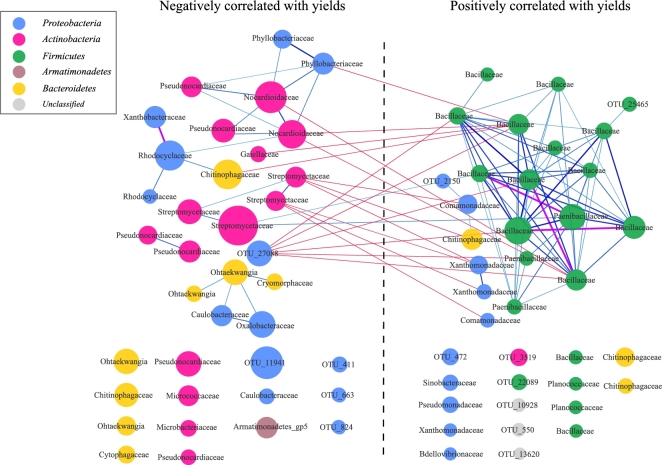
Co-occurrence network of marker OTUs in foxtail millet rhizoplane microbiota. The enrichment direction of marker OTUs was tested by Spearman's rank correlation test (adjusted *P* < 0.05). Negative and positive correlations with yield were plotted. Edges between OTUs were colored according to the Spearman's correlation coefficients (cc > 0.8; purple), between 0.6 and 0.8 (light to dark blue), or cc < 0.4 (red). OTUs annotated to families are colored according to phylum.

Surprisingly, bacteria positively correlated with yields formed a complex network with strong Spearman's correlations, while bacteria negatively correlated with yields showed a simple network with weak connections (Fig. 5). Bacillaceae negatively correlated with Nocardioidaceae, Phyllobacteriaceae, Chitinophagaceae, and Gaiellaceae; Comamonadaceae and Xanthomonadaceae negatively correlated with Streptomycetaceae (Fig. 5). Our results demonstrated that potentially beneficial bacteria tended to co-occur in the root zone of foxtail millet, while non-beneficial bacteria did not form a close correlation.

### Functional prediction and comparison between rhizosphere and rhizoplane microbiome

**Figure 6:**
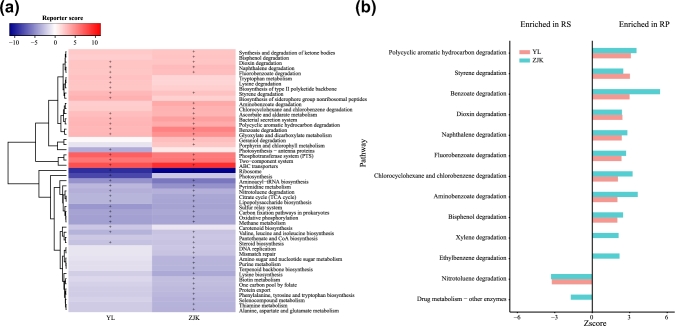
KEGG pathways enriched in rhizoplane or rhizosphere soil samples. (**a**) The relative abundances of pathways were compared between rhizoplane to rhizosphere soil samples from Yangling and Zhangjiakou, respectively. Pathways with a significant difference in reporter score (<1.7, blue, enriched in rhizosphere soil; >1.7, red, enriched in rhizoplane) were retained. +Reporter scores >2.3 or <2.3 are shown in the map. (**b**) Compartment-specific enrichment of xenobiotics biodegradation pathways in rhizoplane and rhizosphere. The reporter scores of pathways greater than 1.7 or lower than –1.7 were plotted. BS: bulk soil; RP: rhizoplane; RS: rhizosphere; YL: Yangling; ZJK: Zhangjiakou.

We predicted the functional profiling of the foxtail millet root microbiome with the PICRUSt package [18]. Rhizosphere and rhizoplane microbiota enriched different Kegg Orthology (KO; KEGG, RRID:SCR_012773) pathways, indicated by reporter scores [19], shown in Fig. 6a. In both Yangling and Zhangjiakou, 12 specific KO pathways were significantly enriched in the rhizoplane microbiome compared with its corresponding rhizosphere (Z-score ≥ 2.3, adjusted *P* < 0.01) (Fig. 6a). The phosphotransferase system (PTS) was significantly elevated in the rhizoplane microbiome (Fig. 6a), which could potentially facilitate rhizoplane bacteria actively absorbing degraded simple sugars (e.g., glucose, mannose, fructose, etc.) from plant root exudates and deposits [20]. Besides, the ABC transporter system was also elevated in the rhizoplane microbiome, which may contribute to the complex exchange of molecules, amino acids, vitamin B12, or iron complex, from densely accumulated rhizoplane bacteria [21]. Additionally, the 2 component system pathways—quorum-sensing gene (*qseC*), bacterial chemotaxis sensor–related gene *(aer, pilJ)*, surface contact signal–sensing gene (*wspA*), biofilm formation–related gene (*rpfC*), competence factor (*comX*)—were enriched in the rhizoplane. These pathways are responsible for intercellular signaling that coordinates bacterial behavior, host colonization, and stress survival to monitor population density [22–24].

Interestingly, we observed an increased abundance of tryptophan metabolism pathway in the rhizoplane microbiome compared to rhizosphere (Fig. 6a). Indole-3-acetic acid (IAA) is a common product of L-tryptophan metabolism produced by many plant growth–promoting rhizobacteria (PGPR) [25, 26]. We found all 4 pathways of IAA biosynthesis: the indole-3-pyruvate (IPyA), indole-3-acetamide (IAM), tryptamine (TAM), and indole-3-acetonitrile (IAN) pathways.

We found that xenobiotics biodegradation and catabolism pathways were mainly present in the rhizoplane microbiome (Fig. 6a). Nine out of 13 sub-pathways were enriched in the rhizoplane microbiome in Yangling and Zhangjiakou (Z-score ≥ 1.7, adjusted *P* < 0.05), while the nitrotoluene degradation pathway was specifically enriched in rhizosphere bacteria (Z-score ≤ –1.7, adjusted *P* < 0.05) (Fig. 6b). Our results showing the prevalence of metabolic pathways corresponding to KEGG category “xenobiotics biodegradation and metabolism” suggest that the foxtail rhizoplane bacterial microbiome may be selected for enzymes capable of adapting to degrade anthropogenic chemicals, such as insecticide and herbicide, and other environmental contaminants that may be widespread in the soil of the targeted regions [27, 28]. Alternatively, the millet microbiota may utilize aromatic compounds from host roots as growth substrates [29, 30]. Previous studies reported that many soil-dwelling bacteria, including Nocardia, Streptomyces, Rhodococcus, and Arthrobacter in the order Actinomycetales, are capable of degrading a wide range of stable xenobiotics like polycyclic aromatic hydrocarbon, benzoate, chlorophenols, and so on [31–35]. In our study, the Actinomycetales, including Nocardia and Streptomyces, were enriched in rhizoplane samples from Yangling and Zhangjiakou (Wilcoxon signed rank test, *P* < 0.01) and may contribute to this functional pattern of the biodegradation of xenobiotics.

Taken together, our results provide important knowledge on the interaction between foxtail millet and its root commensal bacteria. Host plants “influence” bacteria by root exudates with a variety of organic molecules; in return, root bacteria may benefit plant health and productivity in multiple ways, such as producing important plant hormones or degrading harmful chemicals.

## Discussion

We defined the taxonomic structure of the foxtail millet root microbiota, mainly comprising Acidobacteria, Actinobacteria, Bacteroidetes, Firmicutes, and Proteobacteria. Bacterial alpha diversity decreased from foxtail millet rhizosphere to rhizoplane microbiota. These findings were consistent with reports in other plant species such as *Arabidopsis thaliana* [6, 8], maize [2], rice [3], barley [4], grapevine [36], and soybean [37], indicating that the foxtail millet root bacteria follow the general rule of microbiota establishment.

In this study, both CAP and principal coordinates analysis (PCoA) demonstrated that microhabitat was the major factor affecting the bacterial microbiome, rather than geographic locations. Our result was consistent with the findings reported by Lundberg et al. [6], in which the microhabitat was the most important factor driving root bacterial community composition. However, our result was different from the field study of rice, which suggested that the geographic location was a larger source of variation than soil structure and might be a major determining factor shaping the composition of the root microbiome in a setting where the distance between planted locations was up to ∼125 km [3]. Although the distance between Yangling and Zhangjiakou was about 1000 km and the soil types of the 2 fields were obviously different (the soil in the Yangling cropping field was loessal soil, and in Zhangjiakou it was cinnamon soil; this information could be found in a database called Sciences Database of the Chinese Academy of Science [38]), the largest source of the variation of microbial structure was attributed to the rhizospheric compartmentalization. Our result indicated that the foxtail millet was the major factor that drove the assembly of the root-associated microbiome.

We analyzed the association between grain weight of foxtail millet and the corresponding rhizoplane bacterial microbiota using methods similar to those applied in human-associated studies [39, 40]. Through this process, we found that grain weight of foxtail millet could be predicted with explicable variance of 31% (in test data, 29.3%) using abundance of only 75 bacterial OTUs as information. Although 31% was not a high measure of association, it may be considered a reasonable and significant result as it is widely accepted that apart from environmental factors like biological factors and abiotic factors, crop productivity is mainly influenced by plant genotype. Since crop productivity is associated with multiple genes, it is more difficult to identify those key genes/single nucleotide polymorphisms by using genome-wide association study (GWAS), compared to some traits that are associated with a single gene. So far there are no publications showing how the genes determine the foxtail millet yield. The importance of the root-associated microbial community, as the major representative of biological factors, for plant growth and development has been widely recognized [41, 42]. Of the positive markers, 3 main positive genera markers comprised of *Bacillus, Falsibacillus*, and *Paenibacillus*, in which many bacteria were reported with a characteristic that functions as a biocontrol against soil-borne pathogens or with the capability of secreting auxin to promote plant growth [43, 44]. Interestingly, we found that positive marker OTUs showed a strong and complex correlation network, while negative marker OTUs showed a more loose and simple network (Fig. 5). In addition to yield traits, most of these marker OTUs show a positive correlation with growth traits such as main stem width, and top second leaf width (Fig. S3). Our results indicate that cooperative microbial interactions may play critical roles in microbial assembly of the plant microbiome and may benefit plant growth and development. We found that a large number metabolic pathways related to nutrient uptake, environmental responses, and density control were enriched in the microbiota of the root surface, while many of metabolic pathways related to carbon fixation and amino acid synthesis were enriched in the microbiota of the rhizosphere (Fig. 6). The roots of plants excrete 10–44% photosynthetically fixed carbon, which may serve as an energy source, signaling molecules or antimicrobials for soil microorganisms [45], fitting the hypothesis that plants actively shape their root microbiome by root exudates and other rhizodeposits stimulating and/or inhibiting various microbes.

Taken together, our work has systematically characterized the root bacterial microbiota of foxtail millet and identified potential beneficial root bacterial and genomic pathways, which will provide a basis for the application of beneficial root bacteria for agricultural improvement. So, isolation and functional verification of root microbes is necessary for future work, especially for marker species. On the other hand, we will perform metagenomic sequencing of root samples, which will provide more precise qualitative and quantitative functional information of the root microbes than predicted data. It will help further our understanding of this microecosystem in the root zone.

### Potential implications

In this work, we characterized bacterial OTUs’ composition in the root zone using more than 1000 foxtail millet cultivars from 2 well-separated geographic locations. The large sample size allowed us to capture the bacterial diversity in rhizosphere and rhizoplane compartments and assess the existence of a core rhizoplane microbiome for foxtail millet. The data collection serves as an important basis for studying the association of microbial organisms in the foxtail millet root zone with host phenotypes. Large-scale association analysis identified the potential beneficial bacteria correlated with plant high productivity using methods similar to those applied in human-associated studies [39, 40]. The methods for deciphering the association between root microbiome and plant phenotype are applicable to other crops. A better understanding of the various interactions between microbes and the host plant may serve as a valuable knowledge foundation for bio-fertilizer development in sustainable agriculture. Isolation and functional identification of the potentially beneficial bacteria are necessary for future work. Metagenomics combined with quantitative functional genomic approaches like transcriptomics, proteomics, and metabolomics will provide deep insights into microbial interactions and help us fully understand the microecosystem in the root zone.

## Materials and Method

### Microbiome sample collection and plant trait examination

All of the samples of and around foxtail millets (*S. italica*) were collected from the natural fields in September 2013 at Yangling agricultural hi-tech industrial demonstration zone (34°16^΄^18^″^ N/108°4^΄^59^″^ E, Shanxi, China) and Zhangjiakou (40°36^΄^35.49^″^ N/114°56^΄^36.39^″^ E, Hebei, China) using a modified method suggested by Bulgarelli et al. [8]. Three individuals from each millet cultivar species were randomly selected. The roots of 3 millet plants were harvested and shaken to remove the loosely adhering soil particles. The adjacent soil layers (around 1 cm thick) on the roots’ surface were manually separated and collected into a 15-ml Falcon tube as the rhizosphere compartment. The full roots designated for rhizoplane collection were also placed into a 15-ml Falcon tube after cleaning the adjacent soil. In each field, 8 of the bulk soil samples were also collected from an unplanted site far away from the millets. All of the samples were immediately stored at –20°C and transported back to our laboratory. In the laboratory, the whole roots from 3 plants were cut into small sections of 2–3 cm; 0.3 g of these root sections was pooled into a 2-ml tube containing 1.5 ml of sterile PBS-S buffer and washed on a shaking platform for 20 minutes at 25 r/s to remove microbes that tightly adhered at the root surface. Roots were removed, and the washing buffer was subjected to centrifugation for 5 minutes at 1200 rpm. The resulting pellet was kept as a rhizoplane compartment. In total, 1219 foxtail millet cultivars in Yangling and 214 paired cultivars in Zhangjiakou were selected in this study. The details about foxtail millet cultivars and sample types are provided in Table S1. Twelve traits about growth and crop yield, such as top second leaf length and width, main stem height, main stem width, panicle length of the main stem, panicle diameter of the main stem, fringe neck length, panicle weight of the main stem, grain weight per plant, hundred kernel weight, spikelet number of the main stem, and grain number per spike, were recorded for foxtail millets from both geographical sites according the previous study (see Table S2 for details) [14, 46].

### DNA extraction and sequencing for 16S rRNA genes

DNA was extracted from all samples using the PowerSoil DNA isolation kit following the manufacturer's protocol (MO BIO Laboratories, QIAGEN Inc., USA). General universal primers (519F [CAGCMGCCGCGGTAATWC] and 907R [CCGTCAATTCMTTTRAGTTT]) were used to amplify the V3-V5 hypervariable regions of the 16S rRNA gene. The library construction and sequencing on the Ion Torrent PGM platform (BGI-Shenzhen, Shenzhen, China) were carried out as described by Pylro et al. [47].

### Read processing and OTU construction

Reads were processed using the UPARSE pipeline (v. 1.0; UPARSE, RRID:SCR_005020) [48]. Data processing included quality filtering, trimming all sequences to 210 bp, and de-replication. We performed OTU clustering based on 97% pairwise identity along with filtering the chimeras using the UPARSE algorithm, which resulted in a total of 29 076 OTUs. The original reads were assigned back to their OTUs using the USEARCH global alignment algorithm [49], generating the OTU table file. The nonbacterial and mitochondrial OTUs were removed, and taxonomic annotation was performed with the RDP classifier against the RDP database [50]. OTUs whose relative abundance was higher than 0.01% in at least 1 sample were used to generate the profile. In total, 16 109 OTUs in 2882 samples, with an average of 34 264 reads per sample (range = 2013–154 617), were used for subsequent analyses.

### Diversity analysis

Within-sample diversity was calculated for each sample using the Shannon and Chao1 indices via the alpha_diversity.py script in QIIME (v. 1.9.1; QIIME, RRID:SCR_008249) [51] from the final OTU table. Rarefaction curves were calculated using the alpha_rarefaction.py script. The OTU table was normalized by the cumulative sum scaling (CSS) method [52]. Weighted and unweighted UniFrac [53] distances between samples were calculated from the normalized OTU table. A PCoA plot to visualize the differences among groups of samples was performed based on the weighted and unweighted UniFrac distances. To assess the influence of the different factors on the beta diversity, a canonical analysis of principal coordinates (CAP) was also performed using the function capscale() from the R Package vegan v. 2.3.3. The variation and significance of factors were calculated by the function anova.cca() with by = “term” using 999 permutations.

Community similarity and dispersion between sites are integral aspects of β diversity. The difference in microbial community structure between rhizoplane and rhizosphere from each cultivar was tested using two methods: the Sørensen–Dice index and Morisita's overlap index [54, 55]. The Sørensen–Dice index is a statistic used for comparing the similarity of 2 samples only considering the presence or absence of taxa, which could be calculated:

\begin{equation*}{C_S} = \frac{{2{s_{12}}}}{{{s_1} + {s_2}}},
\end{equation*}

where *s*_1_is the number of OTU species in rhizoplane sample, *s*_2_ is the number of OTU species in rhizosphere sample, and *s*_12_ is the shared OTU species of each pair of rhizoplane and rhizosphere samples.

The Morisita-Horn's index of overlap emphasizes differences in the most prevalent taxa between samples, which could be calculated:

\begin{equation*}{C_{mH}} = \frac{{2\mathop \sum \nolimits_{i = 1}^{{s_{12}}} {p_{i1}}{p_{i2}}}}{{\mathop \sum \nolimits_{i = 1}^{{s_1}} p_{i1}^2 + \mathop \sum \nolimits_{i = 1}^{{s_2}} p_{i2}^2}}\ ,
\end{equation*}

where *p_ij_* is the relative abundance of OTU and the definition of *s*_1_, *s*_2_, and *s*_12_ is as above.

These measures of community similarity between samples provide an assessment of the uniqueness of host plant–associated communities. The significance of the index value between Yangling and Zhangjiakou was detected using the Wilcoxon signed rank test with *P* < 0.05.

### Core OTUs

OTUs with occurrence frequency greater than 80% in the rhizosphere, rhizoplane, or soil samples from 1 geographical site were referred to here as “common OTUs.” Thus “common OTUs” in Yangling and Zhangjiakou were obtained. To identify OTUs with quantitative differences between rhizosphere and rhizoplane samples, the Wilcoxon signed rank test was implemented among common OTUs based on the OTU table, with relative abundance considered separately. The *P* values of these tests were corrected for multiple testing using the Benjamini and Hochberg (BH) method [56]. The common OTUs enriched in rhizoplane samples (adjusted *P* < 0.01) were then defined as “core OTUs”.

### Association study

Two methods were used to assess the association of phenotypes of foxtail millets with their rhizoplane microbial communities of Yangling. PERMANOVA was performed using the function adonis() in the R package vegan v. 2.3.3 and the permuted *P* value was obtained based on 999 permutations for the growth and yield traits. While mantel test which was implemented as mantel() in the R package ecodist v1.2.9, was used to identify correlations between microbial community and phenotypes. The Significance of traits was determined according to the adjusted *P* values (<0.05) obtained by both methods after being corrected by the BH method. Correlations between all of phenotypic traits were calculated using Spearman's rank correlation and are shown with a color gradient denoting Spearman's correlation coefficients using R package corrplot (v. 0.77). The block color dented statistical significance with adjusted *P* < 0.05 (Benjamini-Hochberg, Spearman's rank correlation test).

Random forest regression (R package’random ForestSRC’2.0.7) was used to regress the normalized microbiota profiling in rhizoplane of foxtail millet against their grain weight using modified parameters (ntree = 1000 and nodesize = 15), as was done previously by Subramanian et al. [39]. Actually, 709 of 1013 samples of Yangling were randomly selected to train the predicted model, and the other samples were used to test the fitness of it. The 839 OTUs with an occurrence frequency higher than 30% and that were significantly positively or negatively correlated with the grain weight of foxtail millet (BH-adjusted *P* < 0.05, Spearman's rank correlation test) served as input data. A 10-fold cross-validation method was used to determine the optimal OTU set correlated with grain weight. Ranked lists of OTUs in order of random forests reporting feature importance scores were achieved based on the increase in mean square error of grain weight prediction over 100 iterations of the algorithm. The 75 marker OTUs were chosen based on the minimum average cross-validation mean squared errors, which were obtained from 5 trials of the 10-fold cross-validation. The random forest model based on these 75 OTUs was then applied to the test samples (*n* = 304) to predict the grain weight of foxtail millet, which led to a good fit. The training and testing set samples were divided into continuous groups according to grain weight, respectively. The heat maps of both sets based on the profiling of these 75 OTUs were generated using R package gplots v. 3.0.1.

The direction of enrichment of these OTUs was identified by Spearman's rank correlation test (adjusted *P* < 0.05 by BH method). Spearman's correlation coefficient was also calculated between any 2 marker OTUs according to their abundance in the rhizoplane samples of Yangling. The co-occurrence network was visualized by Cytoscape 3.3.0 (Cytoscape, RRID:SCR_003032).

### Microbial function prediction

Microbial function prediction was performed using the PICRUSt software [18]; 60% OTUs were picked using a closed-reference OTU picking protocol (QIIME 1.9.1) against the greengenes database pre-clustered at 97% identify (GG 13.5). The obtained OTU abundance table was normalized by 16S rRNA copy numbers, and metagenomic functions prediction was executed by predict_metagenomes.py, which multiplies normalized OTU abundance by predicted functional trait abundance to produce a table of KO numbers (rows) by samples (columns).

The KEGG function statistics was executed as described by Feng et al. [57]. Briefly, differentially enriched KOs were identified using a 1-tail Wilcoxon rank sum test and the BH correction method. The differentially enriched KEGG pathways were identified according to their reporter score [19] from the Z-scores of individual KOs. A reporter score of Z ≥ 1.64 (95% confidence according to normal distribution) could be used as a detection threshold for significantly differentiating pathways.

## Availability of supporting data and materials

All raw sequencing data have been deposited in the EBI Sequence Read Archive under the BioProject accession number PRJEB16061. Further supporting data can be found in the *GigaScience* respository, *Giga*DB [58].

## Additional files

Fig. S1: Species richness of root-associated microbial communities. (a and b) A rarefaction curve based on the observed value of the chao1 index and OTU numbers was used to depict the sequencing depth and OTU content from rhizoplane and rhizosphere soil samples separately. (c and d) The different gradients of the species diversity and richness of 3 compartments from foxtail millets are shown with violin plots. *P* values come from Kruskal–Wallis tests. BS: bulk soil; RP: rhizoplane; RS: rhizosphere; YL: Yangling; ZJK: Zhangjiakou.

Fig. S2: CAP analysis of the total data reveals that microbiomes vary by rhizocompartments. (a and b) CAP analysis was performed using weighted and unweighted UniFrac metric constrained to compartments and conditioning on cropping site and other technical factors. (c and d) CAP analysis constrained to cropping site and conditioning on compartments and other technical factors. Each dot represents each sample's coordinate on constrained PCoA1. BS: bulk soil; RP: rhizoplane; RS: rhizosphere; YL: Yangling; ZJK: Zhangjiakou.

Fig. S3: (a) Pairwise correlations between 12 traits of foxtail millet. Correlation plot shows positive and negative relationships between healthy phenotypes of foxtail millets from Yangling. Colored blocks indicate statistical significance of *P* < 0.05. If there were no significant correlations between the traits, the blocks are blank. (b) Spearman's correlations between marker OTUs and other traits, such as panicle weight of main stem, panicle diameter of main stem, and top second leaf width. OTUs annotated to families are colored according to phyla; red: positive correlation; blue: negative correlation. +Adjusted *P* < 0.01. ^*^Adjusted *P* < 0.001.

Table S1: Information on bulk soil, rhizosphere soil, and rhizoplane samples in Yangling and Zhangjiakou.

Table S2: Growth and yield phenotypes of foxtail millets in Yangling.

Table S3: OTUs profiling table including reads count and taxonomy information.

Table S4: Average relative abundance of bacterial phyla detected in root compartments and bulk soil samples from Yangling and Zhangjiakou. BS: bulk soil; RP: rhizoplane; RS: rhizosphere; YL: Yangling; ZJK: Zhangjiakou.

Table S5: Permanova result using weighted and unweighted Unifrac distance matrix to define the effect of various factors on microbial community of field foxtail millets (Unifrac dist. ∼ Compartment^*^ Location).

Table S6: OTUs with occurrence frequency between 80% and 100% in the rhizoplane and rhizosphere soil samples from 1 geographical site. The OTUs enriched in rhizoplane samples (*P* < 0.01) were defined as “core OTUs” of foxtail millets. BS: bulk soil; RP: rhizoplane; RS: rhizosphere; YL: Yangling; ZJK: Zhangjiakou.

Table S7: Permanova and Mantel test detecting the association of phenotypes of foxtail millets with their rhizoplane microbial communities of Yangling.

## Abbreviations

BH: Benjamini and Hochberg; BS: bulk soil; CAP: canonical analysis of principal coordinates; KEGG: Kyoto Encyclopedia of Genes and Genomes; KO: Kegg Orthology; OTU: operational taxonomic unit; PCoA: principal coordinates analysis; PERMANOVA: permutational multivariate analysis of variance; PICRUSt: Phylogenetic Investigation of Communities by Reconstruction of Unobserved States; QIIME: Quantitative Insights Into Microbial Ecology; RDP: Ribosomal Database Project; RP: rhizoplane; RS: rhizosphere; YL: Yangling; ZJK: Zhangjiakou.

## Competing interests

The authors declare that they have no competing interests.

## Funding

This work was funded by BGI-Shenzhen, The National Natural Science Foundation of China (U1301252), a Strategic Priority Research Program of the Chinese Academy of Sciences Grant (XDB11020700), and a Priority Research Program of the Chinese Academy of Science Grant (QYZDB-SSW-SMC021).We also want to thank Shenzhen Key Laboratory of Environmental Microbial Genomics and Application, BGI-Shenzhen, Shenzhen, China, for their support.

## Author contributions

Tao Jin, Yayu Wang, Jin Xu, Yang Bai, Huanming Yang, Jian Wang, and Xun Xu established the concept of the study. Guang Liu, Jin Xu, Yuzhen Li, Jing Xu, Haiyan Chu, Gengyun Zhang, Guohua Du, Houbao Zhang, Hongfeng Zou, Haifeng Zhang, Jinying Li, and Jingwang Wan collected and processed samples. Yueying Huang, Pengfan Zhang, Jin Xu, Xin Liu, Honggang Jiang, Guang Liu, Dechun Lin, Yunzeng Zhang, Zhuye Jie, and Suisha Liang performed bioinformatics analyses. Tao Jin, Yayu Wang, Jin Xu, and Yueying Huang performed analysis of data. Tao Jin, Yayu Wang, and Yang Bai wrote the draft. Nian Wang, Karsten Kristiansen, Liang Xiao, Huijue Jia, and Guangyi Fan reviewed and edited the manuscript. Tao Jin, Yayu Wang, Yueying Huang, and Jin Xu contributed equally to the study. All authors have discussed the results and read and approved the contents of the manuscript.

## Supplementary Material

gix089_Supplementary_Data
