# Erratum to: Taxonomic structure and functional association of foxtail millet root microbiome

**DOI:** 10.1093/gigascience/giy099

**Published:** 2018-11-19

**Authors:** Tao Jin, Yayu Wang, Yueying Huang, Jin Xu, Pengfan Zhang, Nian Wang, Xin Liu, Haiyan Chu, Guang Liu, Honggang Jiang, Yuzhen Li, Jing Xu, Karsten Kristiansen, Liang Xiao, Yunzeng Zhang, Gengyun Zhang, Guohua Du, Houbao Zhang, Hongfeng Zou, Haifeng Zhang, Zhuye Jie, Suisha Liang, Huijue Jia, Jingwang Wan, Dechun Lin, Jinying Li, Guangyi Fan, Huanming Yang, Jian Wang, Yang Bai, Xun Xu

In the original, published version of “Taxonomic structure and functional association of foxtail millet root microbiome,” by Tao Jin et al., several changes requested during the proof stage were not implemented. Those changes have now been implemented and the corrected article is available in *GigaScience*, Volume 6, Issue 10.

The Publisher regrets these errors.

Jin T, Wang Yayu, Huang Y. Taxonomic structure and functional association of foxtail millet root microbiome. *GigaScience* 2017 Oct 1;6(10):1–12. doi: https://doi.org/10.1093/gigascience/gix089.

