# Supplementary material for: Taxonomic structure and functional association of foxtail millet root microbiome
Source: Gigascience. 2017 Sep 5;6(10):1–12. doi: 10.1093/gigascience/gix089 (PMC7059795; doi:10.1093/gigascience/gix089)
Supplement: gix089_Supplementary_Data [file gix089_supplementary_data.zip › Table S5.docx]

|  | Weighted UniFrac Distance | | | Unweighted UniFrac Distance | | |
| --- | --- | --- | --- | --- | --- | --- |
| Source | SS | %  Explained | *P* value | SS | % Explained | *P* value |
| Compartment | 14.788 | 32.53 | <0.0001 | 39.02 | 9.9 | <0.0001 |
| Location | 3.29 | 7.23 | <0.0001 | 20.33 | 5.16 | <0.0001 |
| Compartment:Location | 0.814 | 1.79 | <0.0001 | 3.82 | 0.97 | <0.0001 |
| Residuals | 26.575 | 58.45 |  | 330.96 | 83.97 |  |
| Total | 45.466 |  |  | 394.14 |  |  |
