# Supplementary material for: Taxonomic structure and functional association of foxtail millet root microbiome
Source: Gigascience. 2017 Sep 5;6(10):1–12. doi: 10.1093/gigascience/gix089 (PMC7059795; doi:10.1093/gigascience/gix089)
Supplement: gix089_Supplementary_Data [file gix089_supplementary_data.zip › Table S6.docx]

| Location | Sample Type | 80% | 100% |
| --- | --- | --- | --- |
| YL | RP | 683 | 18 |
|  | RS | 1022 | 26 |
|  | Shared | 520 | 9 |
|  | Core_RP | 329 | 15 |
| ZJK | RP | 1038 | 65 |
|  | RS | 1184 | 125 |
|  | Shared | 819 | 46 |
|  | Core_RP | 456 | 51 |
