# Supplementary material for: Taxonomic structure and functional association of foxtail millet root microbiome
Source: Gigascience. 2017 Sep 5;6(10):1–12. doi: 10.1093/gigascience/gix089 (PMC7059795; doi:10.1093/gigascience/gix089)
Supplement: gix089_Supplementary_Data [file gix089_supplementary_data.zip › Table S7.docx]

| **Location** | **YL** | | |
| --- | --- | --- | --- |
| **Traits** | **Sample No.** | **Mantel test adjusted *P* value** | **Permanova adjusted *P* value** |
| Top second leaf length | 1015 | 0.7720 | 0.0060 |
| Top second leaf width | 1015 | 0.0408 | 0.0015 |
| Main stem height | 1018 | 0.2355 | 0.0015 |
| Main stem width | 1017 | 0.0060 | 0.0015 |
| Fringe neck length | 1017 | 0.2143 | 0.0338 |
| Panicle diameter of main stem | 1017 | 0.0300 | 0.0015 |
| Panicle length of main stem | 1017 | 0.2143 | 0.0015 |
| Panicle weight of main stem | 1019 | 0.0080 | 0.0015 |
| Grain weight per plant | 1020 | 0.0060 | 0.0015 |
| Hundred kernel weight | 1017 | 0.3893 | 0.2720 |
| Spikelet number of main stem | 1018 | 0.5568 | 0.0015 |
| Grain number per spike | 1015 | 0.7720 | 0.0027 |
