# Supplementary figures and images for: Taxonomic structure and functional association of foxtail millet root microbiome
Source: Gigascience. 2017 Sep 5;6(10):1–12. doi: 10.1093/gigascience/gix089 (PMC7059795; doi:10.1093/gigascience/gix089)

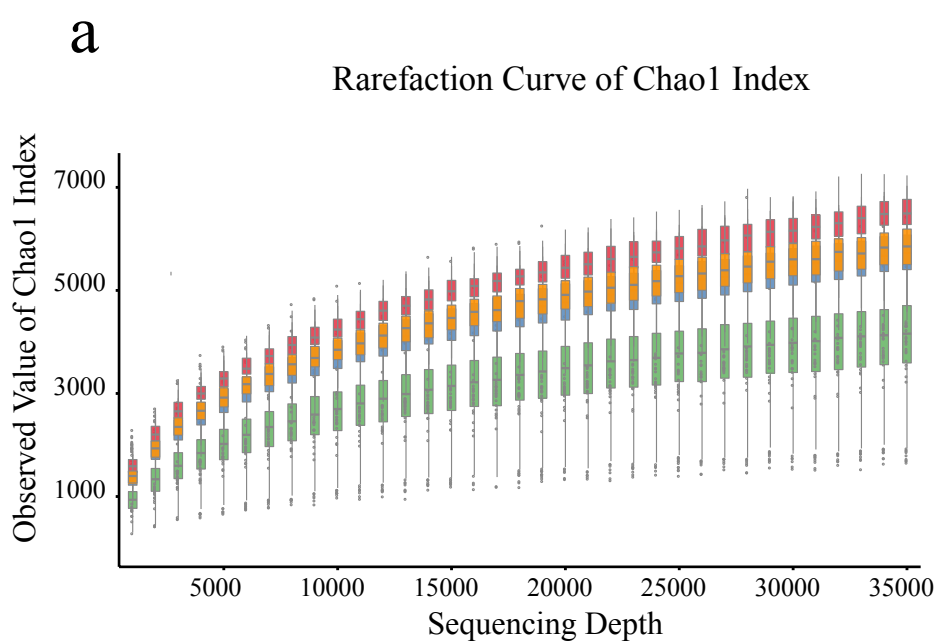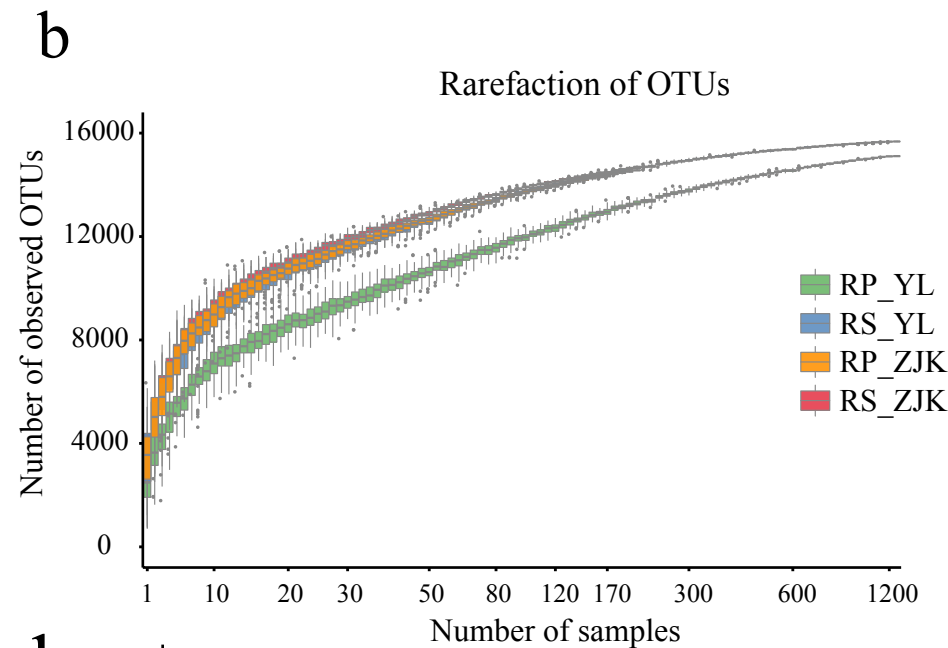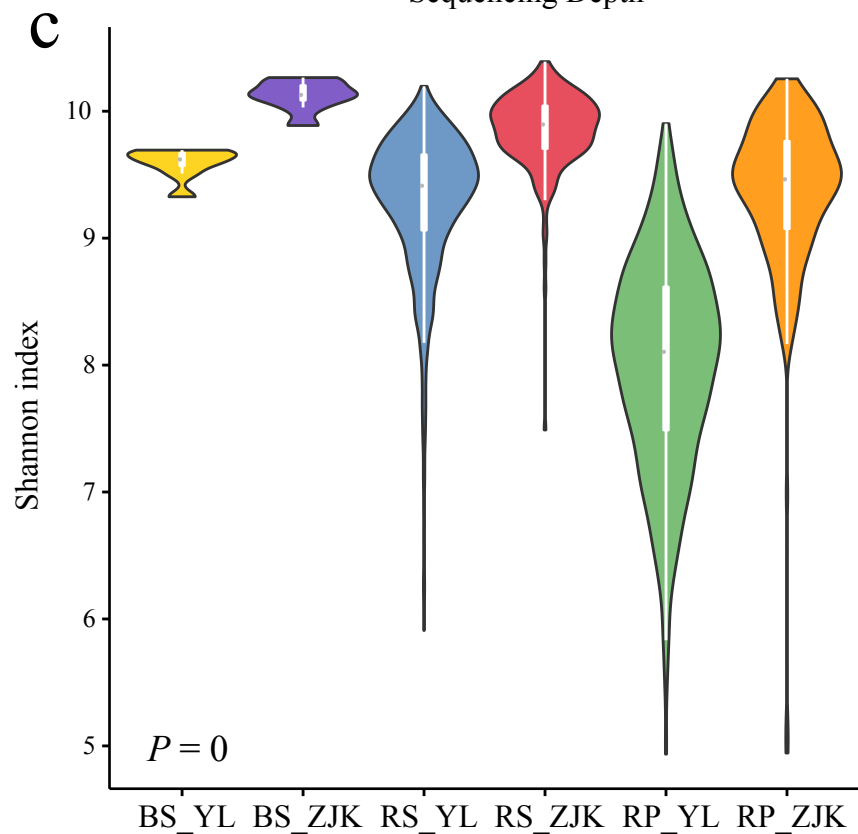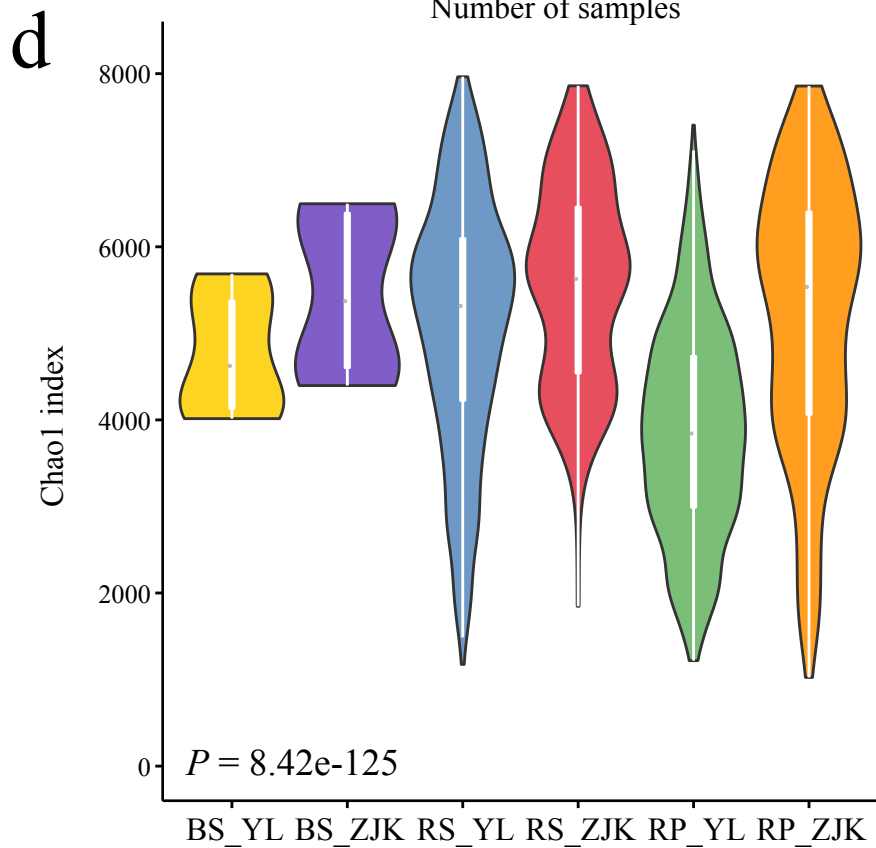

Supplement: gix089_Supplementary_Data [file gix089_supplementary_data.zip › Figure S1.pdf]

# Weighted UniFrac

**a**

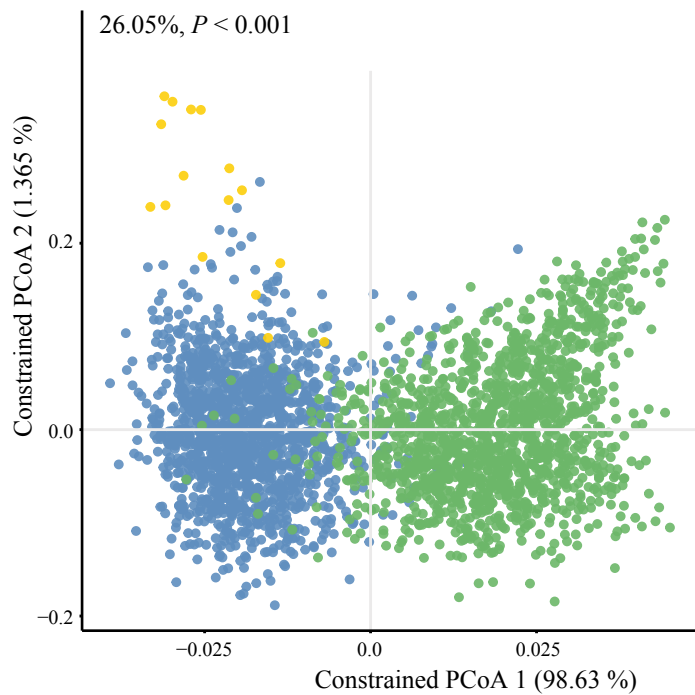

# Unweighted UniFrac

**b**

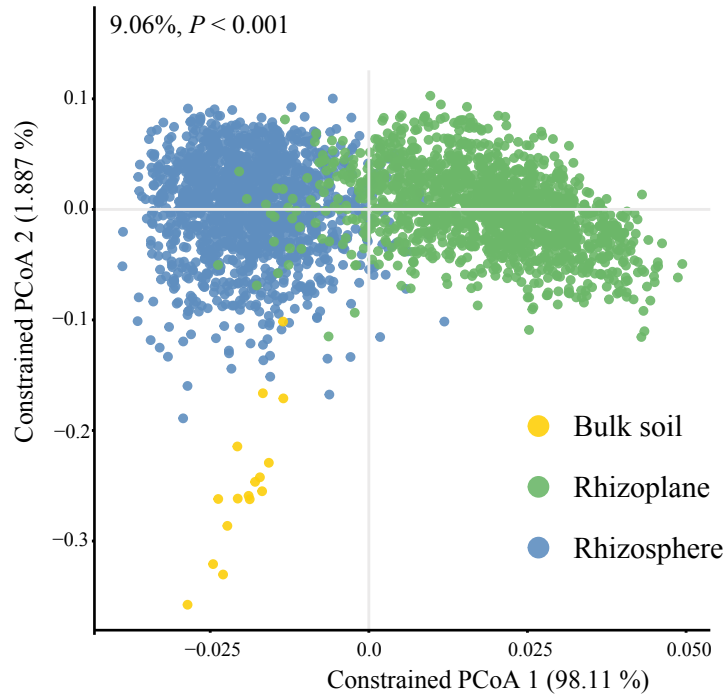

**c**

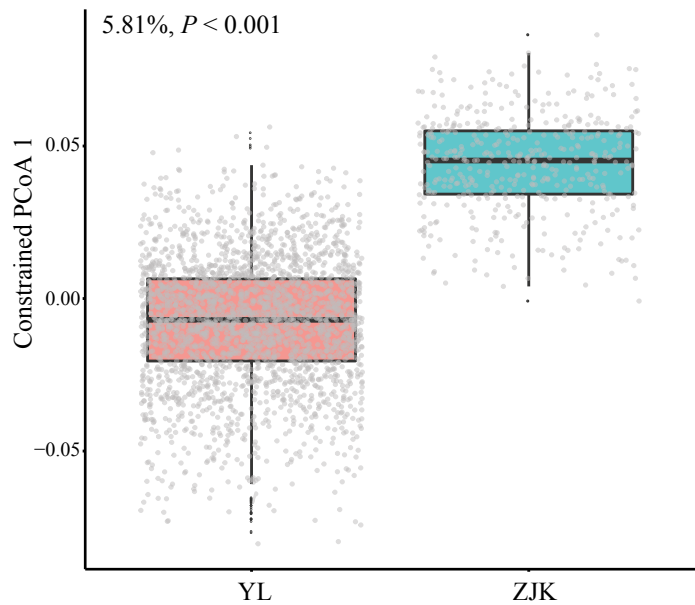

**d**

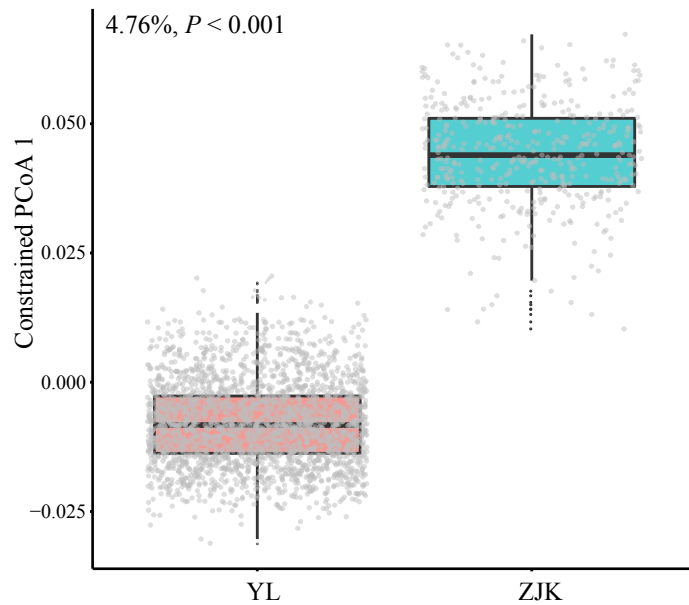

Supplement: gix089_Supplementary_Data [file gix089_supplementary_data.zip › Figure S2.pdf]

a

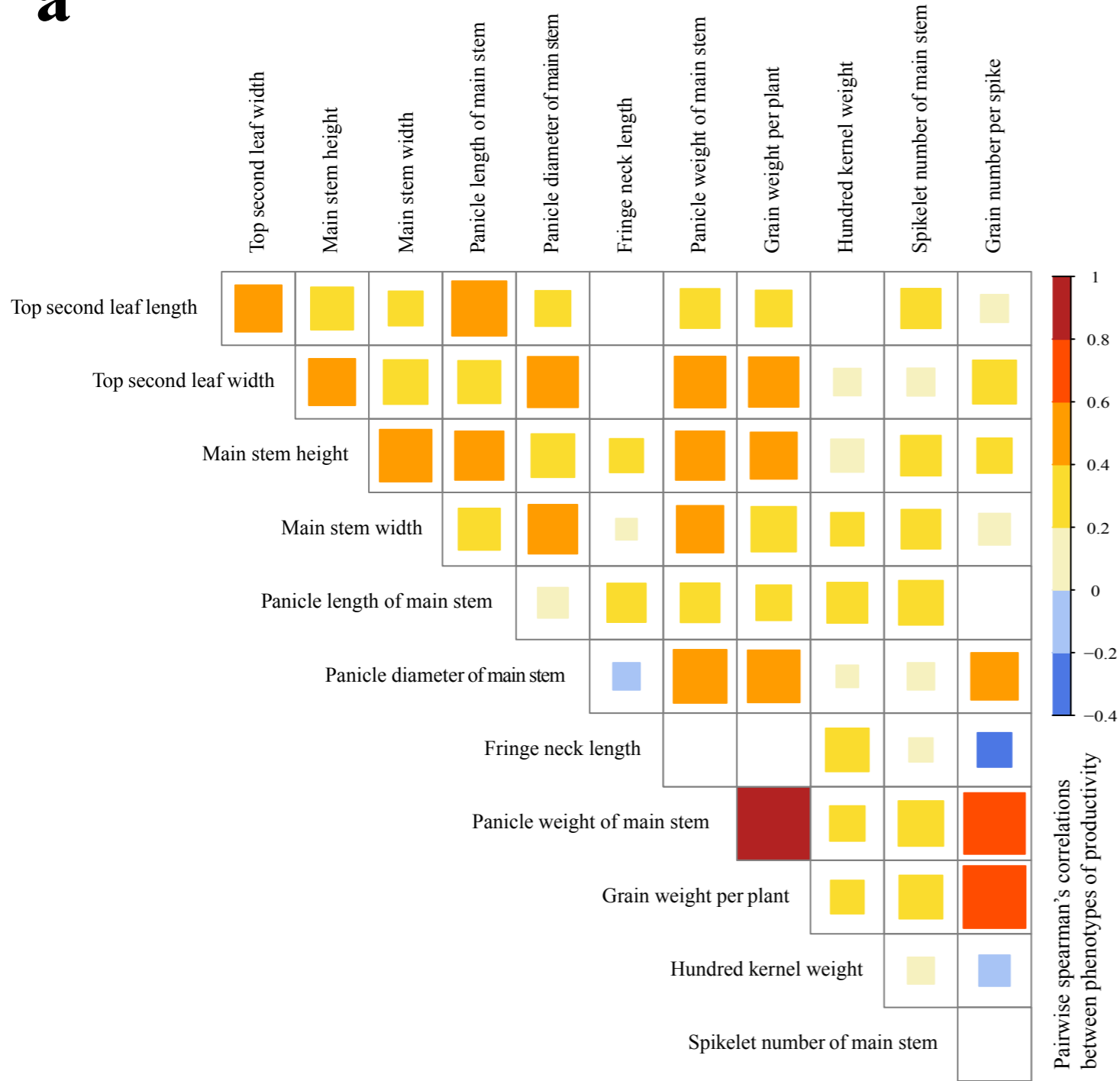

b

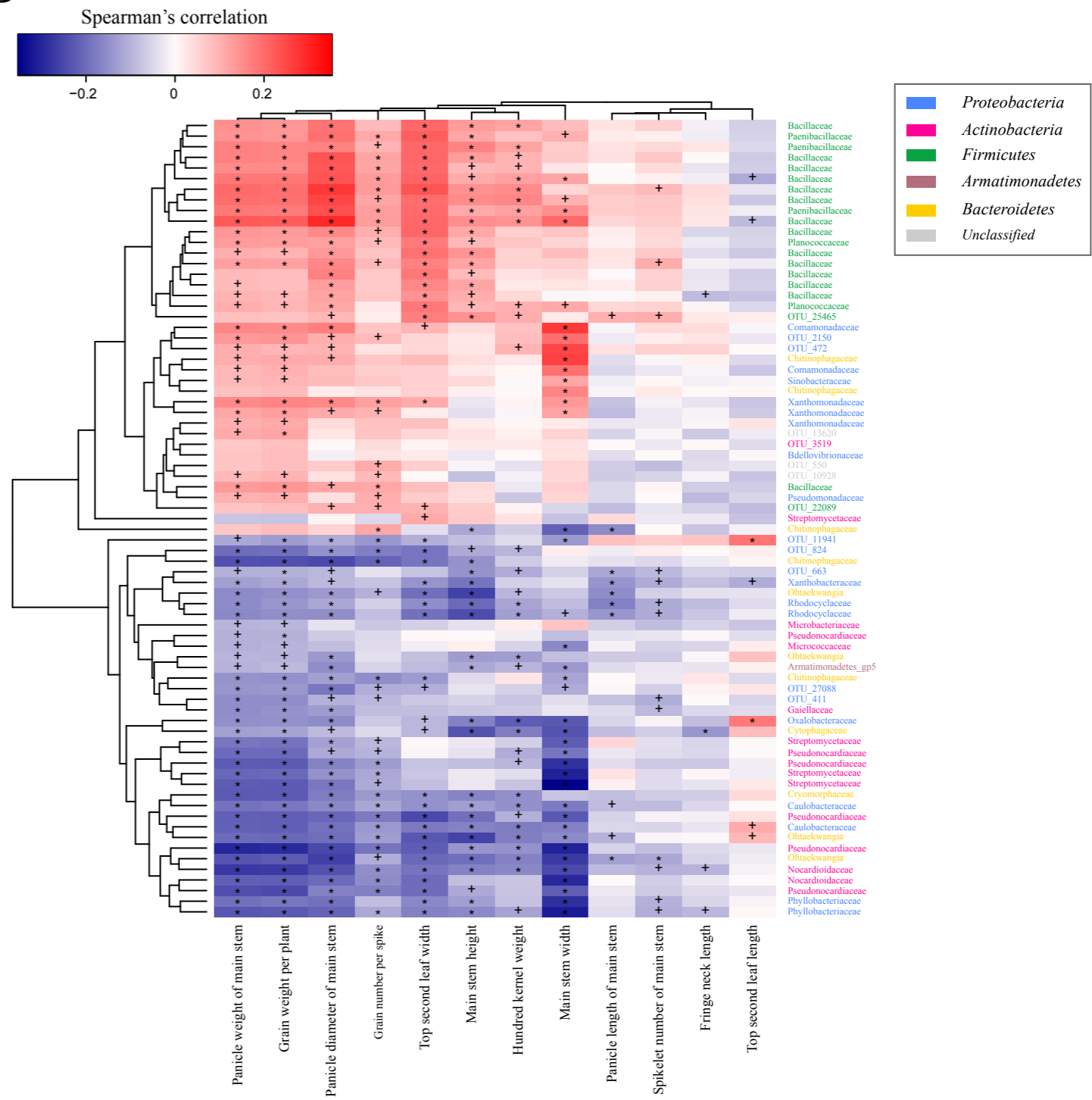

Supplement: gix089_Supplementary_Data [file gix089_supplementary_data.zip › Figure S3.pdf]
